# Damage-softening constitutive model of palm fiber reinforced concrete based on weibull distribution and its correction method

**DOI:** 10.1371/journal.pone.0325602

**Published:** 2025-06-13

**Authors:** Qinghua Zhan, Xiaole Huang, Zhitao Huo, Feiting Yi, Huijuan Bo, Jing Jing, Guanzhi Luo, Jun Dai

**Affiliations:** 1 Changsha General Survey of Natural Resources Center, China Geological Survey, Hunan, China; 2 Xiangyang Polytechnic, Hubei, China; 3 Hubei Water Resources Technical College, Hubei, China; Auckland University of Technology, NEW ZEALAND

## Abstract

Based on the Weibull random distribution of the micro-unit strength of fiber reinforced concrete, the parameters are introduced to describe the micro-unit strength of concrete reasonably, and it is used to establish a damage-softening constitutive model that can reflect the whole process of fiber reinforced concrete damage with the Drucker-Prager yield criterion. On this basis, according to the stress-strain curves of concrete with different palm fiber content and cement content, the functional relationship is discussed among the parameters with palm fiber content and cement content of concrete damage-softening model based on Weibull distribution, then a more realistic concrete damage- softening constitutive model is established with the modified model parameters. To consider the dynamic characteristics of micro-elements in palm fiber concrete during loading-unloading, a modified linear regression model parameter solving method is proposed. By comparing the experimental results with the calculated values, the rationality of the constitutive model and the parameter solution method is verified. The constitutive model proposed in this paper can well reflect the stress-strain relationship and damage evolution process of plam fiber reinforced concrete under different mix ratios, which can provide reference for the engineering application of brown fiber concrete.

## Introduction

In the process of treating the bare rock slope formed by excavation, it is not only necessary to meet the requirements of slope strength and stability, but also to use plam fiber reinforced concrete for ecological protection [1–5]. Plam fiber reinforced concrete is a composite material composed of brown fiber, cement, soil, special additives and organic materials, the slope spray layer formed by it has a certain strength and is not easy to produce cracks, which has strong anti-erosion ability, especially suitable for steep rock slope protection and ecological restoration projects. At present, a lot of research has been carried out on the mechanical properties and constitutive model of plam fiber reinforced concrete. For example, some researchers have carried out experimental research on the performance of concrete under special conditions, and studied the influence of CFRP, steel tube, polypropylene and polyvinyl alcohol fibers on the performance of concrete [6–10]. Other scholars have described the macroscopic mechanical properties of plam fiber reinforced concrete through tests, and analyzed the influence of fiber content, length and cement content on the properties of concrete, which showed that plam fiber reinforced concrete is strain softening, and the strength is significantly different with different fiber content, length and cement content are significantly different [11–14]. Therefore, it is necessary to explore the mechanical properties and damage constitutive model of plam fiber reinforced concrete under different fiber content and cement content.

The constitutive relationship of fiber-reinforced concrete has always been a key and difficult problem in academic research, and it is particularly necessary to establish a reasonable constitutive model of fiber reinforced concrete with the popularization and application of plam fiber reinforced concrete improvement technology in engineering. At present, there are many constitutive models of fiber reinforced concrete. For example, Babu and Chouksey used data regression analysis method to fit the stress-strain curve obtained from the test, and established the constitutive model of fiber reinforced cohesive soil based on Cambridge model [15]. Notta-Cuvier considered the distribution and orientation of fibers, and established the anisotropic damage model of plam fiber reinforced concrete by homogenization method and stress equilibrium equation [16]. EI-Helou established a UHPC strength model and a multi-axial constitutive model by simplifying the internal fiber orientation of the material and considering the influence of the slip energy consumption of the fiber on the material damage [17]. Koci and Foster established a new elasto-plastic constitutive model of fiber-reinforced material, which considered the effect of distributed fibers embedded in a matrix [18]. The above model promotes the development and application of damage constitutive model to a certain extent. However, to establish a scientific and reasonable damage constitutive model of fiber reinforced concrete, it is necessary to understand not only the macroscopic mechanical behavior of materials, but also the microscopic damage evolution mechanism and law of materials. Based on the randomness of the distribution of defects in fiber reinforced concrete, some scholars combine the continuous damage theory with the statistical strength theory, and establish the damage constitutive model of plam fiber reinforced concrete according to the concept that the strength of the material element obeys the Weibull distribution, which can not only reflect the softening characteristics of the whole process of deformation and failure of plam fiber reinforced concrete, but also reflect the characteristics of the strength changing with the pressure [19–23]. However, because the model does not consider the difference of material strength or model parameters under different fiber content and cement content, it cannot reflect the characteristics of softening and strength of plam fiber reinforced concrete with the change of material content, which limits the rationality and applicability of the model.

In this paper, focusing on the functional relationship between the parameters based on Weibull distribution between the fiber content and cement content, establishing a more realistic damage softening constitutive model of plam fiber concrete by modifying the model parameters. Analyzing the structure and deformation failure characteristics of plam fiber concrete, a modified parameter solution method is proposed. Finally, the experimental results are compared with the calculated values to verify the rationality of the model and parameter solution method. The research results make a breakthrough in the study of the constitutive model of the whole process of deformation and failure of fiber reinforced concrete.

## Damage-softening model of plam fiber reinforced concrete based on Weibull distribution

### Traditional damage-softening constitutive model on Weibull distribution

There are two methods to establish the constitutive relation of plam fiber reinforced concrete by using damage theory, the former is based on the principle of energy equivalence of materials before and after damage, and the latter is based on the principle of strain equivalence of materials before and after deformation [24–27]. Due to the mechanical concept of the second method is more clear, so this paper established the constitutive relationship of plam fiber reinforced concrete damage with J.Lemaitre strain equivalence principle, as follows [28],


[σ*]=[σ]/[σ](1−D)\nulldelimiterspace(1−D)=[C][ε]/[C][ε](1−D)\nulldelimiterspace(1−D) (1)

where, $\left[\sigma^{*}\right]$ is the effective stress matrix, [σ] is the total stress matrix, [C] is the stiffness matrix, [ε] is the strain matrix and *D* is the damage variable. It can be seen that the key to establish the damage structure relationship of plam fiber reinforced concrete is to solve the damage variable *D* from Eq. (1). It is assumed that the plam fiber reinforced concrete is composed of damaged and undamaged elements, and the failure criteria of the elements satisfy,


$$f\left(\sigma^{*}\right)-k_{0}=0$$ (2)

where, $k_{0}$ is the constant related to the cohesion and internal friction angle, and $F=f\left(\sigma^{*}\right)$ represents the micro-element strength. Assuming that P(x) is the probability of damage, then the damage variable is,


$$D=\int_{0}^{f\left(\sigma^{*}\right)}P\left(x\right)dx$$ (3)

It is known that Obtaining the damage variable with Eq. (3), it is necessary to solve the strength $F=f\left(\sigma^{*}\right)$ and damage probability P(x) of the plam fiber reinforced concrete micro-element.

#### Micro-element strength of plam fiber reinforced concrete F.

The form of the microelement strength $F=f\left(\sigma^{*}\right)$ is directly determined by the failure mechanism of the plam fiber reinforced concrete and the form of the failure criterion. At present, the failure criteria have many forms of expression, which used to study mechanics of plam fiber reinforced concrete. Considering that the parameters of the Drucker-Prager failure criterion are simple and applicable, the micro-element strength of plam fiber reinforced concrete is derived based on the Drucker-Prager failure criterion as follows [29],


F=f(σ*)=α0I1+J21/12\nulldelimiterspace2 (4)

where, α0=sinφ/sinφ9+3sin2φ\nulldelimiterspace9+3sin2φ, $\begin{array}{c} \varphi \end{array}$ is the internal friction angle, $I_{1}$ and $J_{2}$ are the first invariant of stress tensor and the second invariant of stress deviator, respectively, and having,


$$I_{1}=\sigma_{1}^{*}+\sigma_{2}^{*}+\sigma_{3}^{*}$$ (5)

$$J_{2}=\frac{1}{6}\left[{\left(\sigma_{1}^{*}-\sigma_{2}^{*}\right)}^{2}+{\left(\sigma_{2}^{*}-\sigma_{3}^{*}\right)}^{2}+{\left(\sigma_{1}^{*}-\sigma_{3}^{*}\right)}^{2}\right]$$ (6)

For the triaxial test, there are $\sigma_{1}=\sigma_{3}$ and $\sigma_{1}^{*}=\sigma_{3}^{*}$, which are obtained by Hooke’s law,


ε1=(σ1*−2νσ3*)/(σ1*−2νσ3*)E\nulldelimiterspaceE (7)

σ2*=σ3*=σ3/σ3(1−D)\nulldelimiterspace(1−D) (8)

σ1*=σ1/σ1(1−D)\nulldelimiterspace(1−D) (9)

Substituting Eqs. (7), (8), and (9) to Eqs. (5) and (6), we obtain,


$$I_{1}=\frac{E\varepsilon_{1}\left(\sigma_{1}+2\sigma_{3}\right)}{\sigma_{1}-2\nu \sigma_{3}}$$ (10)

$$\sqrt{J_{2}}=\frac{E\varepsilon_{1}\left(\sigma_{1}-\sigma_{3}\right)}{\sqrt{3}\left(\sigma_{1}-2\nu \sigma_{3}\right)}$$ (11)

Then substituting Eqs. (10) and (11) to Eq. (4), the micro-element strength of plam fiber reinforced concrete can be obtained as follows,


$$\begin{array}{c} F=\frac{\sin\varphi }{\sqrt{9+3\sin^{2}\varphi }}\times \frac{E\varepsilon_{1}\left(\sigma_{1}+2\sigma_{3}\right)}{\sigma_{1}-2\nu \sigma_{3}}+\frac{E\varepsilon_{1}\left(\sigma_{1}-\sigma_{3}\right)}{\sqrt{3}\left(\sigma_{1}-2\nu \sigma_{3}\right)} \end{array}$$ (12)

It can be seen from Eq. (12), the parameters to be solved are the internal friction angle $\begin{array}{c} \varphi \end{array}$, the Poisson’s ratioν, and the elastic modulus *E*.

#### Damage constitutive model based on Weibull distribution.

Assuming that the strength or damage of the plam fiber reinforced concrete element is subject to the Weibull distribution, the probability density function of the element can be expressed,


$$P\left(F\right)=\frac{m}{F_{0}}\times {\left(\frac{F}{F_{0}}\right)}^{m-1}\times \exp\left[-{\left(\frac{F}{F_{0}}\right)}^{m}\right]$$ (13)

where, *m* and *F*_0_ are the Weibull distribution parameters. Substituting Eq. (13) to Eq. (3), obtaining,


$$D=\int_{0}^{F}P\left(F\right)dF=1-\exp\left[-{\left(\frac{F}{F_{0}}\right)}^{m}\right]$$ (14)

The damage model based on Weibull distribution is,


$$\begin{array}{c} \sigma_{1}=E\varepsilon_{1}\left(1-D\right)+\nu (\sigma_{1}+\sigma_{3}\backslash \backslash ;\;\;\;=E\varepsilon_{1}\times \exp\left[-{\left(\frac{F}{F_{0}}\right)}^{m}\right]+2\nu \sigma_{3} \end{array}$$ (15)

The model parameters are the Weibull distribution parameters *m* and *F*_0_ in Eq. (15). It can be divided into two stages in the loading process with plam fiber reinforced concrete, the first stage will not be damaged and the performance of linear elastic deformation, that is, *D* = 0 in Eq. (15), and the second stage is be damaged and the performance of nonlinear law.

### Modified damage constitutive model based on Weibull distribution

**Fig 1:**
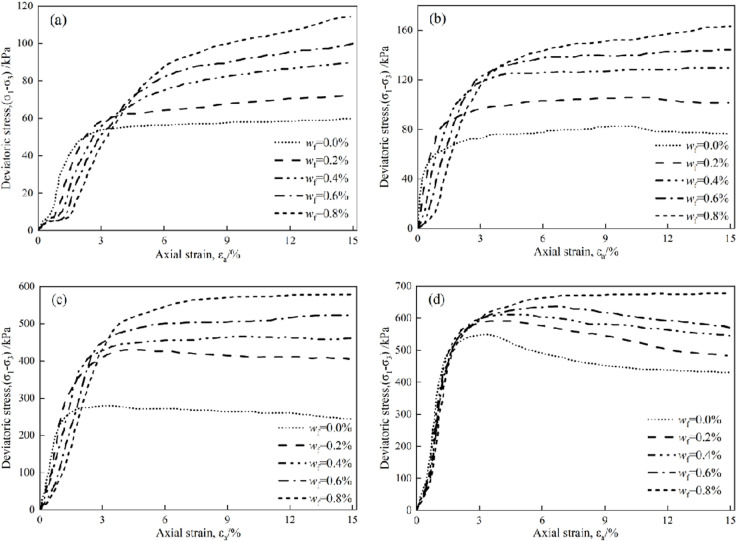
Stress-strain curves of plam fiber reinforced concrete. (a) *x*_c_ = 0%, (B) *x*_c_ = 4%, (c) *x*_c_ = 6%, (a) *x*_c_ = 8%.

The author carried out unconsolidated undrained triaxial compression tests on plam fiber reinforced concrete with different cement content (x_c_) and palm fiber content (w_f_), the test scheme is shown in Table 1, and the test results are shown in Fig 1 [30].

**Table 1:** Mix proportion of sample in unconsolidated undrained triaxial compression tests.

Group	Mix proportion									Test method
	Content of each component of the substrate (%)				Palm fibre (w_f_/ %)					
	Soil	Cement (x_c_)	Organic material	Habitat modifying agent						
I	100	0	6	4	0.0	0.2	0.4	0.6	0.8	Unconsolidated undrained triaxial compression test
II	100	4	6	4	0.0	0.2	0.4	0.6	0.8	
III	100	6	6	4	0.0	0.2	0.4	0.6	0.8	
IV	100	8	6	4	0.0	0.2	0.4	0.6	0.8	

**Fig 2:**
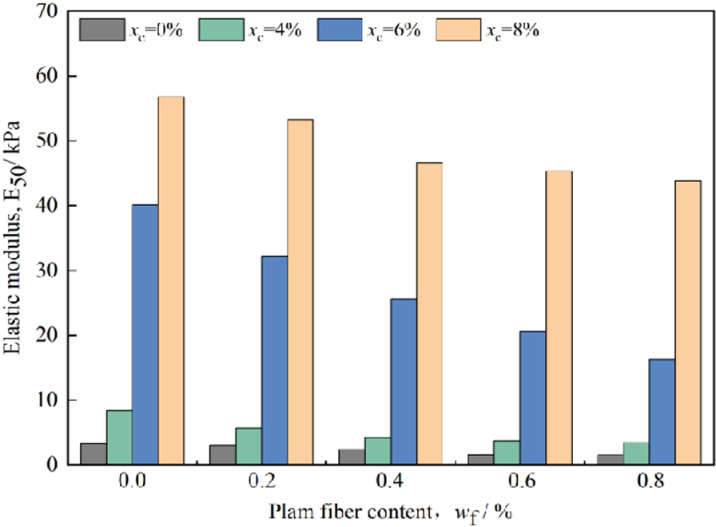
Relationship between elastic modulus of reinforced concrete with palm fiber content and cement content.

E_50_ represents the ratio of stress to strain at 50% of the stress peak strength, and the peak strength under different cement content and palm fiber content is shown in the literature [30], where the elastic modulus E_50_ is determined according to the stress and strain at 50% of the peak strength, and it is found that the elastic modulus E_50_ is not a constant under different mix ratios (Fig 2), indicating that the elastic modulus E_50_ is related to the cement content and palm fiber content.

**Fig 3:**
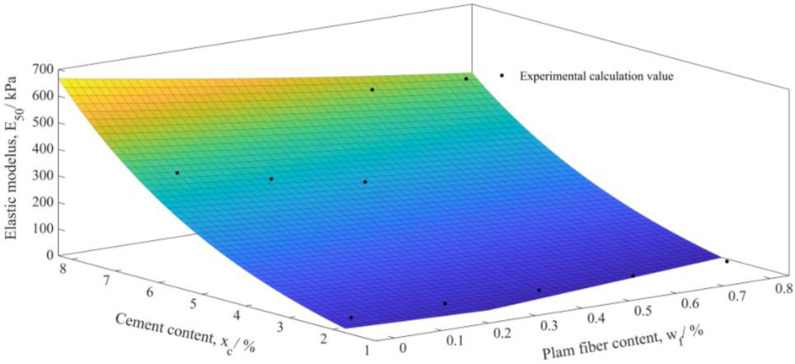
Fitting surface of elastic modulus *E*_50_ of plam fiber reinforced concrete with palm fiber content *w*_f_ and cement content *x*_c_.


By constructing the relationship between the elastic modulus E_50_ and the cement content x_c_ and the palm fiber content w_f_, the damage constitutive model of plam fiber reinforced concrete can be effectively modified. If the cement content x_c_ and palm fiber content w_f_ are taken as x and y coordinates respectively, and the elastic modulus E_50_ is taken as z coordinate, then the (x,y,z) scatter distribution map can be obtained. Matlab is used for exponential surface fitting (Fig 3), the fitting degree is R^2^ = 0.9567, and the fitting function is obtained as follows,

$$E_{50}=-132.3+94.72e^{\left(-0.375w_{f}+0.255x_{c}\right)}$$ (16)

Eq. (16) represents the relationship between the elastic modulus E_50_ and the cement content x_c_ and the palm fiber content w_f_. The modified damage model of plam fiber reinforced concrete can be obtained by substituting Eq. (16) to Eq. (15) as follows,


$$\sigma_{1}=\left[-132.3+94.72e^{\left(-0.375w_{f}+0.255x_{c}\right)}\right]\varepsilon_{1}\times \exp\left[-{\left(\frac{F}{F_{0}}\right)}^{m}\right]+2\nu \sigma_{3}$$ (17)

## The parameter solution method of the correction

### Linear regression method

The solution is usually linear regression to obtain the Weibull distribution parameters m and F_0_, for which Eq. (15) is linearized to get,


−ln[(σ1−2νσ3)/(σ1−2νσ3)Eε1\nulldelimiterspaceEε1]=(F/FF0\nulldelimiterspaceF0)m (18)

Further simplification,


ln{−ln[(σ1−2νσ3)/(σ1−2νσ3)Eε1\nulldelimiterspaceEε1]}=m(lnF−lnF0) (19)

Assumptions,


X=ln{−ln[(σ1−2νσ3)/(σ1−2νσ3)Eε1\nulldelimiterspaceEε1]} (20)

Y=lnF (21)

Then Eq. (19) is,


$$Y=\frac{X}{m}+\ln F_{0}$$ (22)

Assuming that I_1_ and I_2_ are the slope and intercept of linear in Eq. (22), respectively, the Weibull distribution parameters m and F_0_ can be expressed,


$$m=\frac{1}{I_{1}}$$ (23)

$$F_{0}=e^{I_{2}}$$ (24)

### Modified linear regression parameter method

**Fig 4:**
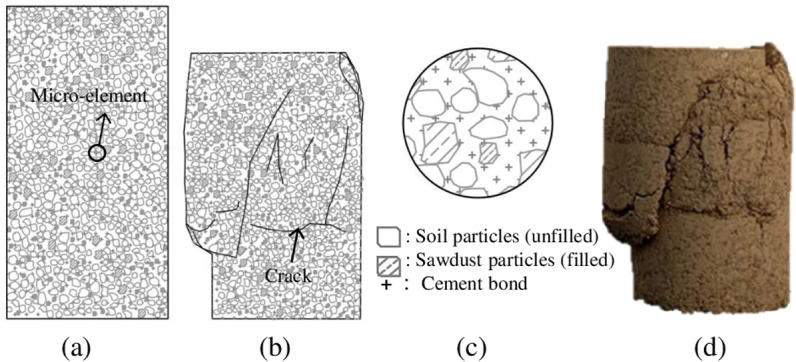
Plam fiber reinforced concrete before and after destruction. (a) before destruction, (B) after destruction, (c) columnar cross section,(d) physical sample.

The rock has the typical characteristics of brittle failure, that is the failure of the performance of local rock cracking and rock fragmentation shedding, which generally will not affect the change of the internal micro-element structure of the rock, so it is usually considered to be constant m and F_0_ in the calculation of the Weibull distribution parameters of the rock. Different from rock, plam fiber reinforced concrete belongs to the semi-rigid material with a porosity of 40%, the cohesion between the particles is insufficient, and the rheological properties of the concrete will be changed due to the incorporation of brown fibers, which caused that its internal micro structure are prone to change under the action of external force (Fig 4), thus the Weibull distribution parameters are also changed before and after the destruction of plam fiber reinforced concrete [31–32]. Therefore, when the linear regression method is used to solve the Weibull distribution parameters, it is necessary to consider the dynamic change characteristics of micro-elements and improve the regular linear regression method.

**Fig 5:**
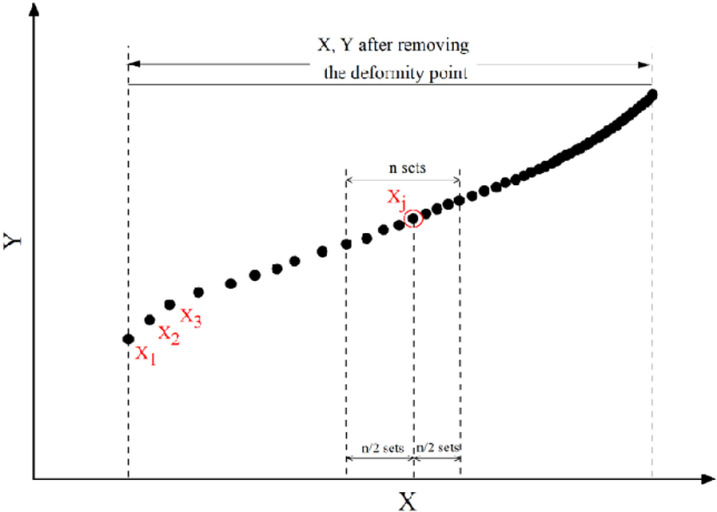
(X,Y) series of data segments.

Considering the dynamic change characteristics of micro-elements in the loading process of plam fiber reinforced concrete, this paper uses the segmented method to solve the slope I_1_ and intercept I_2_ of each segment, and then, it calculates the parameters m and F_0_ according to the slope and intercept of each segment. The specific method is as follows, take X_j_ (j = 1,2,3,......,j is an integer) as the center, take n/2 groups of (X,Y) data (excluding X _j_) to the left, and take n/2 groups of (X,Y) data (including X _j_) to the right. It is calculated according to the actual number of groups when the data is less than n/2 groups (Fig 5), the Weibull distribution function corresponding to each X_j_ point is denoted as m_j_, F_0j_.

**Fig 6:**
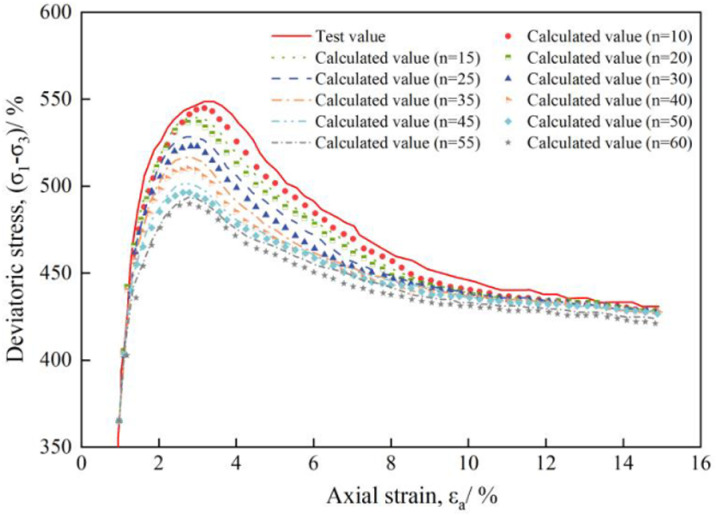
Model calculated value and test value of plam fiber reinforced concrete under different n values (*x*_c_ = 8%, *w*_f_ = 0.0%).

How to determine the value of n? In this paper, it is taking plam fiber reinforced concrete with cement content of 8% and palm fiber content of 0.0 as an example. Considering that when linear regression method is used for calculation, the value of n is at least 10 times the number of independent variables (*x*_i_), then n = 10,15,20,......,60 is taken to analyze the relationship between the model calculation value and the test value under different values of n, as shown in Fig 6.

As can be seen from Fig 6, with the decrease of the n value, the peak value of the model calculation curve gradually increases, and the smaller the error between the calculated value and the test value, that is, when n = 10, the calculated value is in good agreement with the test value. The value of n is best taken from 10 times the number of variables when solving the parameters using linear regression segments. Therefore, the paper selects the segmentation method of n = 10 to calculate the parameters of plam fiber reinforced concrete.

## Validation

In order to verify the modified damage model and parameter solution method, this paper uses the test data under the condition of cement content of 6% for analysis. It is also divided into two stages in determining the calculated value, the first is the initial stage of loading and the material is not damaged, which taking D = 0 to calculate the stress in Eq.(15), where elastic modulus *E* according to Eq. (16). In the second stage, the average slope I_1_ and intercept I_2_ corresponding to each point X_j_ are solved according to the modified parameter solution method, that is, n = 10, and then the parameters m_j_ and F_0j_ are solved corresponding to point x_j_ with Eqs. (23) and (24), respectively. Finally, m_j_ and F_0j_ are substituted into Eq.(17) to obtain the calculated stress value.

**Fig 7:**
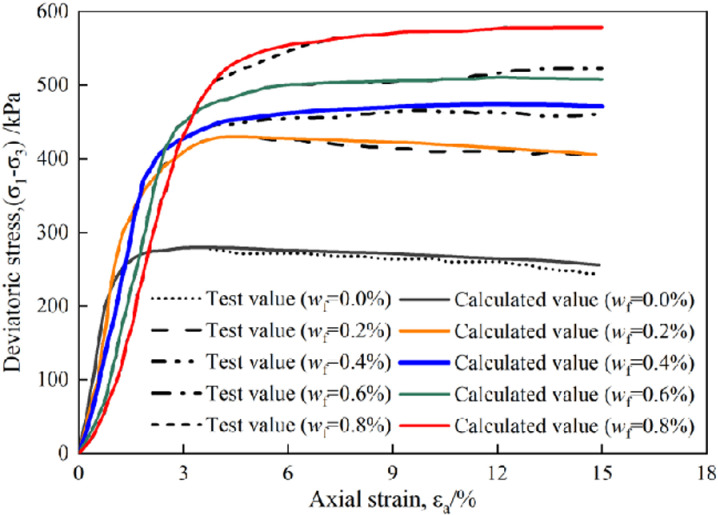
Model calculated value and test value of plam fiber reinforced concrete (*x*_c_ = 6%).

The modified damage model can well describe the stress-strain behavior and fully reflect the softening characteristics of plam fiber reinforced concrete (Fig 7), so the model can be used to analyze and predict the mechanical properties of plam fiber reinforced concrete with different cement content and palm fiber content.

## Conclusions

In this paper, the micro-element strength and micro-element damage of plam fiber reinforced concrete obey the Weibull distribution, introducing the random distribution variable *F* of the micro-element strength of the material, and it deduces the damage evolution equation of the pressure damage of plam fiber reinforced concrete.

(1) The relationship between the elastic modulus *E*_50_ with cement content *x*_c_ and palm fiber content *w*_f_ is discussed basing on the unconsolidated and undrained triaxial test results, and then the damage model is revised in combination with *E*_50_, where a damage model is established that it is more in line with the actual deformation. The model fully reflects that the damage in the destruction process not only changes with the strength, but also is affected by the cement content and palm fiber content in the plam fiber reinforced concrete, the modified damage model is more reasonable.
(2) Plam fiber content and cement content could improve the compressive strength of concrete significantly, plam fiber reinforced concrete showed strain softening with the increase of plam fiber content and cement content. The established model can reflect the whole failure process of plam fiber reinforced concrete under complex stress state, especially the softening characteristics of plam fiber reinforced concrete.
(3) Plam fiber formed a woven network structure in concrete, showing the characteristics of cracks but not broken, which made the process of damage slowly and ductility. Therefore, plam fiber has a certain crack resistance effect on the deformation of concrete.

## Supporting information

S1 Data (XLSX)

S2 Data (XLSX)

S3 Data (XLSX)
